# Aeolian abrasion of rocks as a mechanism to produce methane in the Martian atmosphere

**DOI:** 10.1038/s41598-019-44616-2

**Published:** 2019-06-03

**Authors:** E. Safi, J. Telling, J. Parnell, M. Chojnacki, M. R. Patel, J. Realff, N. J. F. Blamey, S. Payler, C. S. Cockell, L. Davies, I. M. Boothroyd, F. Worrall, J. L. Wadham

**Affiliations:** 1 School of Natural and Environmental Sciences, Newcastle University, Newcastle, NE1 7RU UK; 2 School of Geosciences, University of Aberdeen, Aberdeen, AB24 3FX UK; 3 Lunar and Planetary Laboratory, University of Arizona, Arizona, AZ 85721-0092 USA; 4 School of Physical Sciences, Open University, Milton Keynes, MK7 6AA UK; 5 Department of Earth Sciences, University of Western Ontario, Ontario, ON N6A 3K7 Canada; 6 School of Physics and Astronomy, University of Edinburgh, Edinburgh, EH9 3FD UK; 7 School of Geographical Sciences, University of Bristol, Bristol, BS8 1SS UK; 8 Department of Earth Sciences, Durham University, Durham, DH1 3LE UK

**Keywords:** Atmospheric chemistry, Geochemistry

## Abstract

Seasonal changes in methane background levels and methane spikes have been detected *in situ* a metre above the Martian surface, and larger methane plumes detected via ground-based remote sensing, however their origin have not yet been adequately explained. Proposed methane sources include the UV irradiation of meteoritic-derived organic matter, hydrothermal reactions with olivine, organic breakdown via meteoroid impact, release from gas hydrates, biological production, or the release of methane from fluid inclusions in basalt during aeolian erosion. Here we quantify for the first time the potential importance of aeolian abrasion as a mechanism for releasing trapped methane from within rocks, by coupling estimates of present day surface wind abrasion with the methane contents of a variety of Martian meteorites, analogue terrestrial basalts and analogue terrestrial sedimentary rocks. We demonstrate that the abrasion of basalt under present day Martian rates of aeolian erosion is highly unlikely to produce detectable changes in methane concentrations in the atmosphere. We further show that, although there is a greater potential for methane production from the aeolian abrasion of certain sedimentary rocks, to produce the magnitude of methane concentrations analysed by the Curiosity rover they would have to contain methane in similar concentrations as economic reserved of biogenic/thermogenic deposits on Earth. Therefore we suggest that aeolian abrasion is an unlikely origin of the methane detected in the Martian atmosphere, and that other methane sources are required.

## Introduction

The Mars Science Laboratory Curiosity rover has measured background levels of atmospheric methane a metre above the Martian surface of 0.41 ± 0.16 ppb/sol with spikes of up to 7 ppb^1,2^. Ground-based observations suggest larger methane spikes (plumes) with an average peak of 33 ppb and a maximum value of 45 ppb^3^. The UV irradiation of meteoric-derived organic matter within surface sediments appears to be one of the most plausible mechanisms for producing low background levels of methane^4,5^. However, the cause(s) for seasonal changes in methane background levels and methane spikes remain enigmatic. Additional proposed sources of methane include hydrothermal reactions with olivine^6,7^, organic breakdown via meteoroid impact^4,8^, release from gas hydrates^9^, or biological production^6,10^. It has also been suggested that the release of methane from fluid inclusions in basalt during aeolian erosion could release detectable methane to the Martian atmosphere^11^, yet to date, there has been no quantitative estimate of this flux. Further, the potential for the aeolian abrasion of sedimentary rocks to produce the methane concentrations detected by Curiosity and ground-based observations is completely unexplored, despite the presence of abundant sedimentary rocks on the Martian surface, including Gale Crater^12^.

Due to the current lack of significant liquid water, it is likely that aeolian abrasion has been a dominant mechanism of surface weathering on the Martian surface for the last 3 billion years^13^. The average erosion rate on Mars between the Hesperian and present are many orders of magnitude lower than those on Earth, of the range 1 × 10^−5^–0.01 µm yr^−1^ ^14^. More recent examination of HiRISE time-lapse images have suggested far higher rates of local abrasion underneath active sand dunes that can match those in some arid regions on Earth. Inter-dune field abrasion rates of local basaltic bedrock are in the range of 0.1–50 µm yr^−1^ ^15,16^. Importantly, many terrestrial minerals and rocks contain gases trapped in discrete inclusions, fractures or within the grains themselves (intragranularly)^11,17^. This includes not only igneous rocks, such as basalts, but evaporites and mudstones, all of which are exposed on the Martian surface^12,18^. A recent analysis of a range of Martian meteorites has confirmed the presence of methane gas trapped within Martian basalt inclusions, with coincident concentrations of other gases indicating a Martian origin rather than later terrestrial contamination^19^. Methane in terrestrial basalts (and by analogy Martian basalts) most likely derives from a combination of original magmatic methane and methane generated through water-rock interactions at elevated temperatures^19,20^. In contrast, methane preserved within sedimentary deposits formed in surface environments on Earth, typically has a biogenic origin (from the local activity of *in situ* methanogenic bacteria living within the sediments of the primary evaporitic environment) and/or a thermogenic origin (resulting from the thermal alteration of biological organic matter)^21^.

On Earth the release of methane from fluid inclusions is a negligible component of atmospheric methane. This is due to the far greater fluxes of methane from extant biology^21^, anthropogenic sources^21^, thermogenic sources^21^, and to a smaller extent abiogenic sources from active volcanism and hydrothermal activity^21^. On present day Mars, however, there is greater potential for fluid inclusion release to have a significant impact on atmospheric chemistry. This is owing to the combination of far lower atmospheric pressures (approximately 7–10 mbar^22^), meaning the escaped methane would be less diluted, with very low background methane concentrations (sub ppb) in the Martian atmosphere^1,2,19^. Additionally, there is a lack of evidence for present day substantial methane fluxes from biogenic, thermogenic or abiogenic sources^23^.

Here, via unpublished and previously published laboratory measurements, we estimate the potential production of methane in the Martian atmosphere from the release of methane within fluid inclusions via aeolian abrasion.

### Estimation of methane fluxes from aeolian abrasion at varying time-scales

We estimate methane fluxes from aeolian abrasion by combining estimates of a range of current Martian surface abrasion rates (µm yr^−1^) with published and newly determined methane contents (nmol g^−1^) from a range of SNC meteorites and analogue terrestrial rocks. For basalt, we used abrasion rates of 1 × 10^−5^ µm yr^−1^ as measured by Pathfinder to represent average rates since the Hesperian^14^ and assume a tenfold greater rate of abrasion for softer layered sedimentary rocks (e.g. mudstone)^15^. We used an average rate of abrasion of 0.75 µm yr^−1^, as measured from radiometric dating by Curiosity^12^, to represent long term abrasion rates in Gale Crater where Curiosity has observed background methane and methane plumes^2^. A rate of 0.75 µm yr^−1^ is also representative of rates of basalt sand abrasion under actively moving sand dunes^15^. To represent the highest estimates of abrasion in active sand dune fields on vertical rock faces, we used 50 µm yr^−1^ for basalt^16^, and tenfold greater (500 µm yr^−1^) for evaporites and mudstones/shales. The methane contents of Martian and terrestrial basalts, and terrestrial evaporites, minerals (quartz, plagioclase feldspar, magnetite) and mudstones/shales from a combination of published literature and new experiments were determined by a variety of different methods (Supplementary Methods).

We first calculated gas fluxes from aeolian abrasion for a period of one hour, assuming vertical mixing over a 0.5 km atmospheric height. These calculations are relevant for short-term (20 min to 1 hour) *in situ* measurements taken by the Curiosity Rover around Gale Crater^1,2^. We also calculated gas fluxes integrated over 30 sols, assuming vertical mixing over the entire Martian atmospheric column. These calculations are relevant for the formation of larger scale methane plumes^3^ (Supplementary Methods).

### Aeolian abrasion of basalt is unlikely to explain observed methane plumes

**Figure 1:**
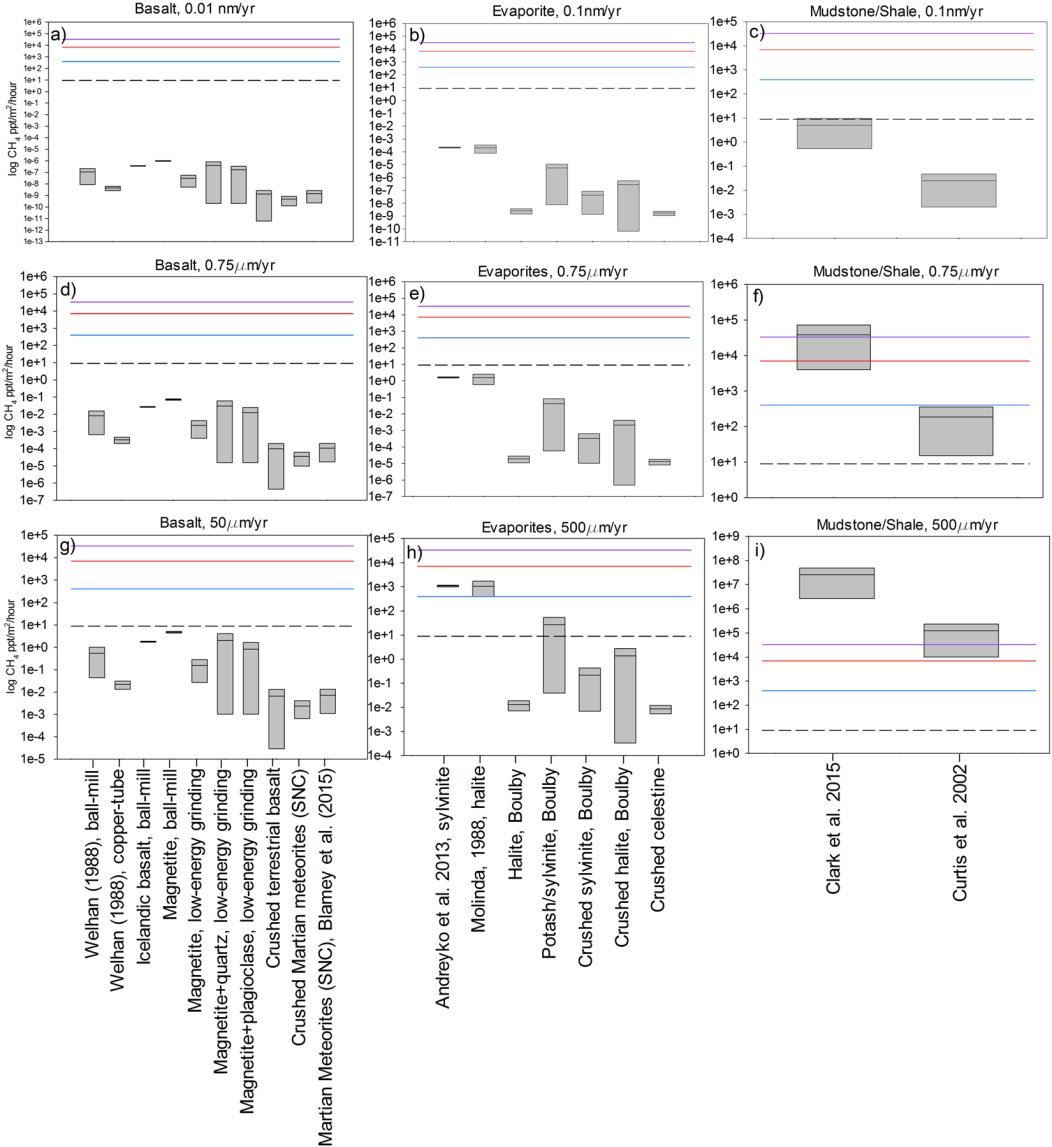
Estimated methane fluxes from the aeolian abrasion of analogue Martian rock samples using a one hour time period and assuming vertical mixing over 0.5 km. A range of abrasion rates from published literature were used to calculate the methane fluxes: (**a**) basalt with an abrasion rate of 1 × 10^−5^ µm yr^−1^, (**b**) evaporites, 1 × 10^−4^ µm yr^−1^, (**c**) mudstone/shale, 1 × 10^−4^ µm yr^−1^, (**d**) basalt, 0.75 µm yr^−1^, (**e**) evaporites, 0.75 µm yr^−1^, (**f**) shale, 0.75 µm yr^−1^, (**g**) basalt, 50 µm yr^−1^, (**h**) evaporites, 500 µm yr^−1^, (**i**) mudstone/shale, 500 µm yr^−1^. The purple line is the average (33 ppb) methane flux of the plume measured by ground-based observations^3^, the red and blue lines are the peak (700 ppb) and average (400 ppb) values of methane measured by the Curiosity rover respectively^2^ and the dashed line represents the methane flux from organic breakdown^5^. The box that represents each sample is bound by the maximum and minimum flux from a range of measurements (see Supplementary Information), and the line situated in the box represents the median value of the fluxes from the samples.

**Figure 2:**
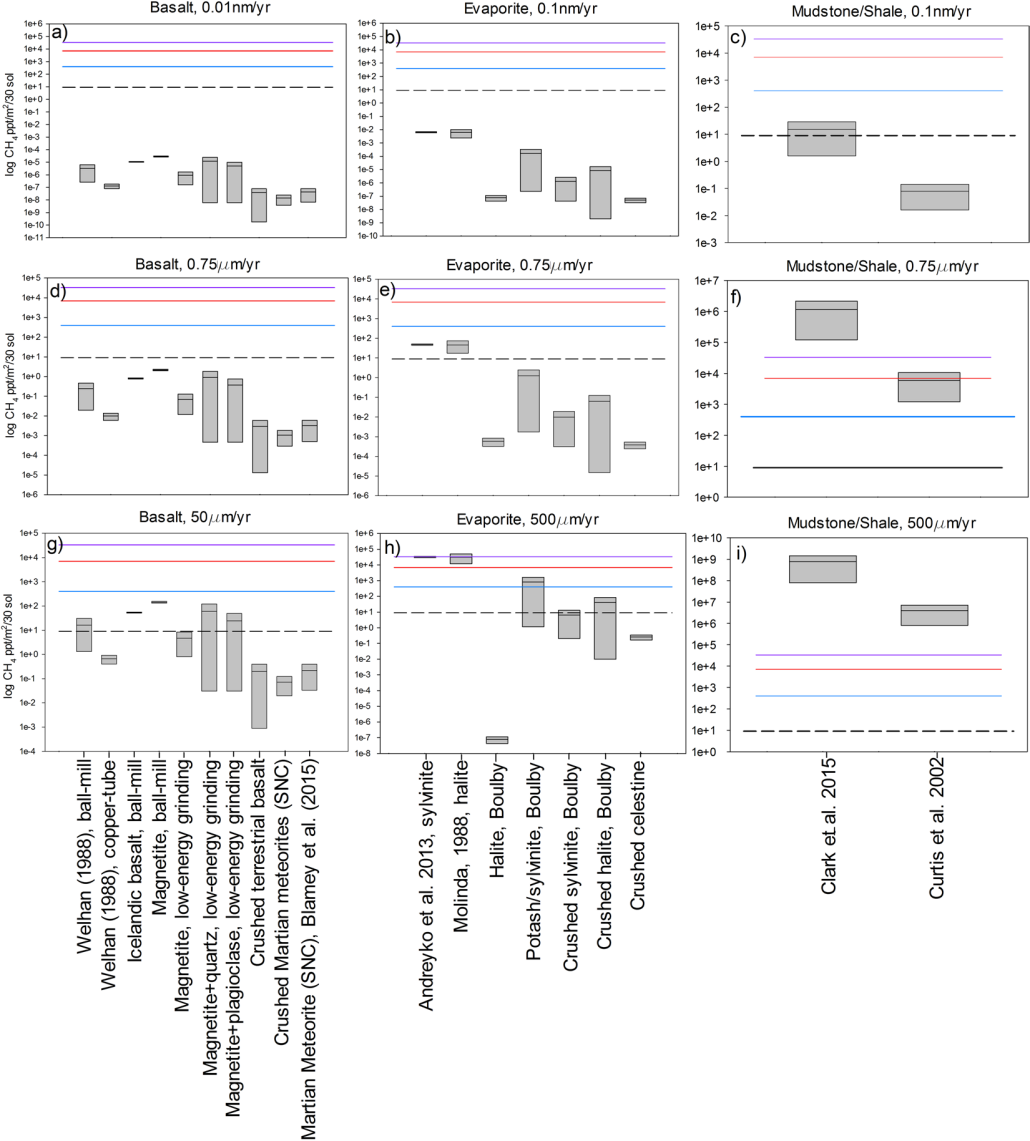
Estimated methane fluxes from the aeolian abrasion of analogue Martian rock samples using a period of 30 sols and assuming vertical mixing over the entire Martian atmospheric column; a – g the methane flux was calculated from: (**a**) basalt with abrasion rate of 1 × 10^−5^ µm yr^−1^, (**b**) evaporites, 1 × 10^−4^ µm yr^−1^, (**c**) shale, 1 × 10^−4^ µm yr^−1^, (**d**) basalt, 0.75 µm yr^−1^, (**e**) evaporites, 0.75 µm yr^−1^, (**f**) mudstone/shale, 0.75 µm yr^−1^, (**g**) basalt, 50 µm yr^−1^, (**h**) evaporites, 500 µm yr^−1^, (**i**) mudstone/shale, 500 µm yr^−1^. The purple line is the average (33 ppb) methane flux of the plume measured by ground-based observations^3^, the red and blue lines are the peak (700 ppb) and average (400 ppb) values of methane measured by the Curiosity rover respectively^2^ and the dashed line represents the methane flux from organic breakdown^5^. The box that represents each sample is bound by the maximum and minimum flux from a range of measurements (see Supplementary Information), and the line situated in the box represents the median value of the fluxes from the samples.

Figures 1a,d,g and 2a,d,g show the methane flux from basalt and Martian meteorite samples using three different abrasion rates over a period of 1 hour and 30 sols respectively. Over a time period of 30 sols, abrasion rates of 1 × 10^−5^ µm yr^−1^ and 0.75 μm yr^−1^ are unable to produce sufficient methane to compete with estimates generated from the breakdown of meteoritic material by UV irradiation^5^. Even the highest abrasion rate of 50 μm yr^−1^ is incapable of producing concentrations of methane above the atmospheric background levels determined by the Curiosity rover (Fig. 1). Our data (Figs 1 and 2) also demonstrate that, when analysed by the same method (crush-fast scan technique), the range of methane contents of terrestrial basalts encompasses that of Martian meteorites. Therefore, it seems unlikely that Martian basalt will have substantially higher methane contents than their terrestrial counterparts, particularly given the potential for incorporation of biogenic carbon into terrestrial basalts via plate tectonic recycling. From our data, we conclude that the aeolian erosion of basaltic-type rocks and derived sand grains on Mars is an unlikely mechanism for the elevated methane concentrations detected by both ground-based observations^3^ and the Curiosity rover^2^ unless there were substantially higher concentrations contained within other types of igneous rocks on Mars (e.g. peridotites^24^).

### The potential formation of methane plumes from the aeolian abrasion of sedimentary rocks

At Gale Crater, the surface geology is dominated by mudstones and sandstones^12^. The mudstones are thought to represent an ancient lake environment with potentially habitable conditions^12^. Clay minerals have also been identified in the east of the Arabia Terra, Nili Fossae and the southeast quadrant of Syrtis Major^3^. These regions represent the potential source area of the larger methane plume identified by ground-based observations. Additionally, sulphate-bearing materials have been detected by visible images obtained by the MRO High Resolution Imaging Science Experiment (HiRISE) and Context Camera (CTX)^5^ and by visible‐near infrared reflectance spectra obtained by the Compact Reconnaissance Imaging Spectrometer for Mars (CRISM) from orbit stratigraphically above Curiosity’s current location^25^. The latter showed an abundance of hydration signatures interpreted as either sulphate-cemented clays or alternating thin beds of clay minerals and sulphates^25^. The detection of sulphates in the Gale Crater and their distribution on Mars as a whole provides evidence for widespread evaporitic environments involving surface to near subsurface aqueous processes extending into the Hesperian^25^.

Our results indicate that the aeolian abrasion of terrestrial analogue sedimentary rocks (evaporites, mudstones/shales) have the potential to produce significantly higher fluxes of methane to the Martian atmosphere compared to basalt. This is due to a combination of higher maximum methane contents and higher susceptibility to abrasion (Figs 1 and 2). For example, using the average abrasion rates of mudstones/shale at Gale Crater (0.75 μm yr^−1^) over a time period of one hour produces, in some cases, more than an order of magnitude greater methane concentrations than the highest methane concentrations detected by Curiosity^2^_._ At an elevated abrasion rate of 500 µm yr^−1^, the most methane-rich evaporites also produce methane above measured atmospheric background levels. While such high rates of abrasion are clearly implausible averaged over the long term, they may be achievable during the abrasion of e.g. vertical faces during short duration elevated abrasion events^16^. On a larger scale over 30 sols, our calculations demonstrate that at an abrasion rate of 0.75 µm yr^−1^, the most methane-rich mudstone/shale terrestrial samples could even provide enough methane to exceed the fluxes required for the formation of larger plumes, as documented in the east of Arabia Terra, Nili Fossae, and the southeast quadrant of Syrtis Major^3^. However, as sedimentary deposits on Mars tend to be localized, even if the vertical methane fluxes were maintained over 30 sols, the concentrations documented in Fig. 2 would be greatly diluted via horizontal mixing^1,26^.

Crucially, however, the relatively high methane fluxes from the aeolian abrasion of sedimentary rocks are obtained by the use (in the absence of other data sources) of terrestrial rock analogues that include organic-rich biogenic/thermogenic deposits. In contrast, analyses of the mudstones at Gale Crater by Curiosity have so far detected organic molecules up to only 24 ppm organic carbon^27,28^ similar to the organic content of a range of igneous Martian meteorites (20 ± 6 ppm^29^), which, as we discuss above, are a highly unlikely source of detectable Martian atmospheric methane. Furthermore, the sum of inorganic carbon gases produced from Curiosity’s evolved gas analyser only suggests a total organic content of up to 2384 ppm; similar to the organic content in fine grained sediments beneath middle portions of the South Pacific Gyre (SPG) region on Earth^30^, rather than the highly productive marine or lacustrine environments responsible for terrestrial economic hydrocarbon deposits^31^. Furthermore, recent gravimetric surveys suggest that Gale Crater sediments have not been buried to sufficient depths to commence the onset of methane generation via significant organic matter thermogenesis^32^. Therefore, it seems highly unlikely that sediments in Gale Crater could contain comparable methane contents to methane-rich terrestrial sedimentary rocks shown in Figs 1 and 2, unless the methane is produced by a very different mechanism.

One such speculative alternative mechanism for methane production is suggested by the presence of features termed ‘hollow nodules’ within the Sheepbed member mudstones at Gale Crater. A possible interpretation of these hollow nodules is they represent ancient gas bubbles formed during authigenic mineral precipitation^33^. Subsequent geochemical modelling has indicated that sufficient hydrogen gas could be produced during authigenic magnetite precipitation to produce these gas bubbles^26^. Combined with CO_2_ this hydrogen could have generated the required redox gradient to drive potential biological methanogenesis^26^. We note, however, that although the majority of any gas might be expected to escape during the drilling process, no elevated methane concentrations were detected during the pyrolysis of Gale Crater sediment associated with the hollow nodules^34^.

Finally, a recent summary of aeolian activity in Gale Crater^35^ demonstrates that elevated aeolian activity has occurred at Gale Crater in the southern summer season over the last several Martian years (between approximately Ls 180-Ls 360). The strong seasonality of sand fluxes at Gale Crater is consistent with observations at other sites on Mars; for example sand fluxes in the Nili Patera dune field in the Northern hemisphere are three times higher during the southern summer compared to winter^36^. Crucially, however, the observed sand activity at Gale Crater does not appear to have a correlation with the observed background methane concentrations detected by Curiosity at approximately Ls 180 or methane spike at Ls 90. This indicates that an alternative source of methane is required to explain the seasonal background changes and isolated higher peaks in methane detected by the MSL Curiosity.

### Production and destruction of methane gas via UV and cosmic irradiation of surface rocks

A significant difference between Martian and terrestrial surface rocks is in their differing exposures to UV and cosmic radiation. Collectively the Earth’s atmosphere and magnetic field absorb a substantial fraction of short-wave solar UV radiation, and deflect charged particles such as galactic cosmic rays and solar energetic particles^37^. In contrast, Martian surface rocks are currently exposed to relatively high levels of UV-C irradiation and cosmic radiation. The shielding depth of the Earth’s atmosphere from ionising radiation is 1000 gcm^−2^^ 33^ compared to Mars’ 16 gcm^−2^. Indeed, it has been suggested that UV photolytic processes could be responsible for the formation of the detected background methane via the breakdown of meteoric/cometary derived organic matter in surface sediments^38^ (see dashed line in Figs 1 and 2). It has been estimated from laboratory experiments that approximately 20% of meteoritic/cometary organic matter in Martian surface sediments could be converted to methane via UV irradiation, although the exact figure will depend on a variety of other factors, including the availability of water and mineral oxidants^5^. This conversion rate would input 64 tonnes of methane into the atmosphere per year, equivalent to an atmospheric column integrated concentration of 2.2 ppbv^5^. However, the penetration depth of UV in rocks is limited to a range of a few microns to less than a millimetre depending on composition^39,40^, and hence unlikely to make a significant impact on atmospheric methane fluxes over existing estimates^4^. Below the UV penetration depth, cosmic ray irradiation (including solar energetic protons and galactic cosmic rays) will typically dominate the alteration of organic molecules in the upper few metres^40^. While studies have examined the effects of cosmic ray irradiation on the alteration of amino acids^33^, there have been no studies reporting the specific effect on methane gas concentrations in representative rocks. However, it has been suggested via analogous studies on the irradiation of organic molecules in the terrestrial crust that, at least over geological periods of time, gamma irradiation could result in the polymerization of methane gas to higher molecular weight and higher C:H ratio compounds, such as polyaromatic hydrocarbons (PAHs)^31^. If by analogy similar polymerization reactions are induced by cosmic ray irradiation of the upper metres of the Martian surface, then the survival of any initial methane gas trapped within fluid inclusions or fractures in rocks might be restricted to regions which have been relatively recently exhumed through e.g. meteorite excavation or scarp retreat^12^. We recommend further experimental studies quantifying the effects of cosmic ray irradiation on methane production and destruction within relevant analogue rock types.

From the data put forward in this paper, we conclude that aeolian abrasion of basaltic or sedimentary rocks on the Martian surface is an unlikely mechanism to produce methane concentrations detected by *in situ* observations from the MSL Curiosity rover and remote ground-based sensing observations. Hence, we suggest that other sources of methane gas must be inferred to explain both the seasonal variations in background atmospheric methane and higher concentration plumes detected on Mars.

## Supplementary information

Supplementary Information

## Data Availability

The datasets generated during and/or analysed during the current study are available from the corresponding author on reasonable request.

## References

[1] Webster CR (2018). Background levels of methane in Mars’ atmosphere show strong seasonal variations. Science.

[2] Webster CR (2015). Mars methane detection and variability at Gale crater. Science.

[3] Mumma MJ (2009). Strong Release of Methane on Mars in Northern Summer 2003. Science.

[4] Moores JE, Schuerger AC (2012). UV degradation of accreted organics on Mars: IDP longevity, surface reservoir of organics, and relevance to the detection of methane in the atmosphere. Journal of Geophysical Research: Planets.

[5] Schuerger AC (2012). Methane from UV‐irradiated carbonaceous chondrites under simulated Martian conditions. Journal of Geophysical Research: Planets.

[6] Formisano V (2004). Detection of Methane in the Atmosphere of Mars. Science.

[7] Sgavetti M (2015). Spectral reflectance characteristics of the Hamar Laghdad hydrothermal sequence, Morocco: Implications for the methane origin on Mars. Icarus.

[8] Hand E (2018). Mars methane rises and falls with the seasons. Science.

[9] Stevens AH, Patel MR, Lewis SR (2015). Numerical modelling of the transport of trace gases including methane in the subsurface of Mars. Icarus.

[10] Tung HC, Bramall NE, Price PB (2005). Microbial origin of excess methane in glacial ice and implications for life on Mars. Proceedings of the National Academy of Sciences of the United States of America.

[11] McMahon S, Parnell J, Blamey NJF (2013). Sampling methane in basalt on Earth and Mars. International Journal of Astrobiology.

[12] Farley KA (2014). *In Situ* Radiometric and Exposure Age Dating of the Martian. Surface. Science.

[13] Bridges NT (2005). Trajectories and energy transfer of saltating particles onto rock surfaces: Application to abrasion and ventifact formation on Earth and Mars. Journal of Geophysical Research: Planets.

[14] Golombek MP, Bridges NT (2000). Erosion rates on Mars and implications for climate change: Constraints from the Pathfinder landing site. Journal of Geophysical Research: Planets.

[15] Chojnacki M, Banks M, Urso A (2018). Wind‐Driven Erosion and Exposure Potential at Mars 2020 Rover Candidate‐Landing Sites. Journal of Geophysical Research: Planets.

[16] Bridges NT (2012). Earth-like sand fluxes on Mars. Nature.

[17] Crawford, M. L. & Hollister, L. S. Metamorphic Fluids: The Evidence from Fluid Inclusions. *New York*, *NY: Springer New York* (1986).

[18] Murchie SL (2009). A synthesis of Martian aqueous mineralogy after 1 Mars year of observations from the Mars Reconnaissance Orbiter. Journal of Geophysical Research: Planets.

[19] Blamey NJF (2015). Evidence for methane in Martian meteorites. *Nature*. Communications.

[20] Welhan JA (1988). Methane and hydrogen in mid-ocean-ridge basalt glasses: analysis by vacuum crushing. Canadian Journal of Earth Sciences.

[21] Whiticar MJ (1999). Carbon and hydrogen isotope systematics of bacterial formation and oxidation of methane. Chemical Geology.

[22] Hess SL (1980). The annual cycle of pressure on Mars measured by Viking Landers 1 and 2. Geophysical Research Letters.

[23] Oehler DZ, Etiope G (2017). Methane Seepage on Mars: Where to Look and Why. Astrobiology.

[24] Kiefer WS (2015). The effects of mantle composition on the peridotite solidus: Implications for the magmatic history of Mars. Geochimica et Cosmochimica Acta.

[25] Milliken, R. E., Grotzinger, J. P. & Thomson, B. J. Paleoclimate of Mars as captured by the stratigraphic record in Gale Crater. *Geophysical Research Letters* **37** (2010).

[26] Tosca NJ (2018). Magnetite authigenesis and the warming of early Mars. Nature Geoscience.

[27] Freissinet C (2015). Organic molecules in the Sheepbed Mudstone, Gale Crater, Mars. Journal of Geophysical Research: Planets.

[28] Eigenbrode JL (2018). Organic matter preserved in 3-billion-year-old mudstones at Gale crater, Mars. Science.

[29] Steele A, McCubbin FM, Fries MD (2016). The provenance, formation, and implications of reduced carbon phases in Martian meteorites. Meteoritics & Planetary Science.

[30] Sutter B (2017). Evolved gas analyses of sedimentary rocks and eolian sediment in Gale Crater, Mars: Results of the Curiosity rover’s sample analysis at Mars instrument from Yellowknife Bay to the Namib Dune. Journal of Geophysical Research: Planets.

[31] Court RW (2006). The alteration of organic matter in response to ionising irradiation: Chemical trends and implications for extraterrestrial sample analysis. Geochimica et Cosmochimica Acta.

[32] Lewis KW (2019). A surface gravity traverse on Mars indicates low bedrock density at Gale crater. Science.

[33] Kminek G, Bada J (2006). The effect of ionizing radiation on the preservation of amino acids on Mars. Earth and Planetary Science Letters.

[34] Stern, J. C. *et al*. Evidence for indigenous nitrogen in sedimentary and aeolian deposits from the Curiosity rover investigations at Gale crater, Mars. *Proceedings of the National Academy of Sciences* 201420932 (2015). 10.1073/pnas.1420932112 PMC4394254 25831544

[35] Baker MM (2018). Coarse Sediment Transport in the Modern Martian Environment. Journal of Geophysical Research: Planets.

[36] Ayoub F (2014). Threshold for sand mobility on Mars calibrated from seasonal variations of sand flux. Nature Communications.

[37] Sinha N, Kral TA (2018). Effect of UVC Radiation on Hydrated and Desiccated Cultures of Slightly Halophilic and Non-Halophilic Methanogenic Archaea: Implications for Life on Mars. Microorganisms.

[38] Keppler F (2012). Ultraviolet-radiation-induced methane emissions from meteorites and the Martian atmosphere. Nature.

[39] Cockell CS, Raven JA (2004). Zones of photosynthetic potential on Mars and the early Earth. Icarus.

[40] Dartnell, L. R. *et al*. Modelling the surface and subsurface Martian radiation environment: Implications for astrobiology. *Geophysical Research Letters* **34** (2007).

