# Supplementary material for: Aeolian abrasion of rocks as a mechanism to produce methane in the Martian atmosphere
Source: Sci Rep. 2019 Jun 3;9:8229. doi: 10.1038/s41598-019-44616-2 (PMC6546745; doi:10.1038/s41598-019-44616-2)
Supplement: Supplementary file 1 — Supplementary Information [file 41598_2019_44616_MOESM1_ESM.docx]

**Supporting Information for:**

**Aeolian abrasion of rocks as a mechanism to produce methane in the Martian atmosphere**

**Safi, E.^1^, Telling, J.^1*^, Parnell, J.^2^, Chojnacki, M.^3^_,_ Patel, M.^4^_,_ Realff, J.^1^, Blamey, N. J. F.^5^, Payler, S.^6^, Cockell, C. S^6^., Davies, L.^7^, Boothroyd, I.M.^8^, Worrall, F.^8^, Wadham, J.L.^7^**

**^1^ School of Natural and Environmental Sciences, Newcastle University, NE1 7RU, UK.**

**^2^ School of Geosciences, University of Aberdeen, AB24 3FX, UK.**

**^3^** **Lunar and Planetary Laboratory, University of Arizona, AZ 85721-0092, US.**

**^4^ School of Physical Sciences, Open University, MK7 6AA, UK.**

**^5^ Department of Earth Sciences, University of Western Ontario, ON N6A 3K7, Canada.**

**^6^ School of Physics and Astronomy, University of Edinburgh, EH9 3FD, UK.**

**^7^ School of Geographical Sciences, University of Bristol, BS8 1SS, UK.**

**^8^ Department of Earth Sciences, Durham University, DH1 3LE, UK.**

***corresponding author,** [jon.telling@newcastle.ac.uk](mailto:jon.telling@newcastle.ac.uk)

**Supplementary Information**

**Supporting text**

**Methods**

**Crush-fast scan technique (basalts, Martian meteorites and evaporites)**

The analysis of trapped gas in fluid inclusions was performed on twenty-seven basalt samples, five Martian meteorites and twenty-five evaporite samples. The experimental procedure and set-up is the same as that described by previous experiments^1^. Briefly, samples were analysed using the crush-fast scan technique, where crushing at room temperature released the gases confined in fluid inclusions (see Supplementary Tables 1, 2 and 3 for total methane released), into a quadrupole mass spectrometer. Each individual crush produced a burst of gas that was analysed. Standard gas mixtures and fluid inclusion standards were used to calibrate the gas composition from the samples. This procedure does not heat the gas to release it which is advantageous as heating can convert carbon containing components into different forms. However, a disadvantage of the method is that only a fraction of the total gas within the sample is likely to be released during the procedure.

**Agate gas tight ball mill method (Icelandic basalt, quartz, feldspar, magnetite)**

An 80 ml gas-tight agate-grinding bowl with 5 × 2 cm diameter agate balls and a Teflon o-ring was used to mill the crushed rock samples via a Fritsch Planetary Mono Mill Pulverisette 6. Prior to rock milling and between different materials, 10 g of sand was milled at 450 rpm for 2 minutes. The sand was cleared from the mill using compressed nitrogen gas and the process repeated until clean.

10 g of various materials (Supplementary Table 3) were weighed into the agate grinding bowl. The grinding bowl was sealed using a gastight agate lid with two Swagelok valves inserted through it; one allowing connection to a vacuum line, and the other fitted with a gastight septa to allow direct sampling of the gases via a syringe and needle. The grinding bowl was then flushed with 5.0 grade Ar (BOC), and the gases evacuated using an Edwards RV5 vacuum pump. This cycle of Ar flushing and evacuation was repeated 5× prior to experimental runs in order to remove any traces of atmospheric contamination. Blanks were analysed after one and thirty minutes to check for any residual contamination or leaks. In successive runs, with new material added for each run, the material was then milled at 450 rpm at times of 1, 2, 5, 10, 15, 20, and 30 minutes. At the end of each run, 5 ml of 5.0 grade Argon was injected via a gastight syringe and needle, then 5ml of headspace gas removed and the gas used to flush a 0.5 ml sample loop on an Agilent 7890A Gas Chromatograph (GC) with an FID detector. Linear calibration curves were prepared by dilution of a certified (± 5%) standard (BOC Special Gases) containing 194.7 ppm CH_4_. The coefficient of variation for CH_4_ was 0.8 % (n = 20).

**Low energy grinding experiments (Magnetite, quartz, feldspar)**

Borosilicate serum vials (100 ml, Wheaton) were washed in a 10% HCl bath for 2 hours, then washed 6 × in deionized water. All vials were furnaced for four hours at 450⁰C to eliminate traces of organic matter. Butyl rubber stoppers (Bellco) were boiled in 1 M NaOH for two hours, rinsed six times with deionised water and dried in an oven (70°C) overnight. 10 to 11 g of different materials (Supplementary Table 4) were placed into serum vials and dried in an oven at 100⁰C for 24 hours to remove water. Note that the grain size of the quartz and feldspar was 250 μm to 2 mm, while a < 63 µm starting fraction of magnetite was used. Each vial was then stoppered and crimp-sealed while still hot. Before the start of experiments, the headspace of each vial was flushed with 5.0 grade Ar by inserting a needle through the bung and evacuating the vial via connection to an Edwards RV5 vacuum pump. The vials were then refilled with 5.0 grade Ar. This cycle was repeated 5 × to remove any residual air contamination.

The vials were placed on an orbital shaker at 0°C. The rotation speed of the orbital shaker was adjusted to give a rotational energy of 6.74 x 10^-4^ J, as defined by:

$RKE=\frac{1}{2}m(r^{2}w^{2})$ (1)

Where m is the mass in kg of the material, r is the radius in mm, and w is the angular speed in radians/s. This is equivalent to an energy input of 0.06 watts/kg of material; similar to the estimated mechanical power input required to initiative sand saltation on Mars^2^.

The headspaces of vials at the end of experiments (35 days) was measured using an Agilent 7890A Gas Chromatograph (GC) with an FID detector, as described above. Results are shown in Supplementary Table 6, normalized both to grams of total material, and grams of magnetite.

Note that these minerals were mixed with quartz and feldspar (see Supplementary Table 6) as it provides a more realistic analogue to the Martian environment than using the pure mineral alone. Also, since quartz is harder than plagioclase you would expect greater magnetite abrasion with quartz – as reflected in the results.

**Dissolution method (halite, potash)**

Weighed amounts of halite or potash were placed into pre-cleaned (see Section above) Wheaton serum bottles. Serum vials were sealed with pre-cleaned (see above) Bellco stoppers, and via a needle evacuated with an Edwards RV5 vacuum pump. 5.0 grade He or Ar was then added by allowing a 50 ml syringe filled with He to equilibrate with the vial to atmospheric pressure. Next, 20ml of helium flushed MILLI-Q™ water was injected into the vials to dissolve the salts. To test the optimum time for salts to be in solution, prior tests were run by monitoring the conductivity of the solution using a Hanna HI9835 conductivity/TDS meter, and measuring the time required for the conductivity measurements to stabilize.

To analyse headspace gases, aliquots of gas were injected either by flushing a 0.5 ml sample loop onto an Agilent 7890A Gas Chromatograph (GC) with an FID detector, or by direct injection (100 μl) directly onto the column of a Thermofisher Trace Gas GC-PDD (Supplementary Table 7). In addition, 10 ml of the headspace gases were diluted in 5.0 grade He within 5L gastight aluminium Tedlar bags, and the δ^13^C-CH_4_ of the gases analysed using a Picarro g2201-i and analyser GC. The accuracy of the δ^13^C-CH_4_ values were checked against a certified CH_4_ standard, diluted with 5.0 Grade He and analysed in the same way as the samples, with a known δ^13^C-CH_4_ of -23.9 ‰ relative to the VDPB standard. Results are summarized in Supplementary Table 8.

**Comparison of extraction methods**

All methods used or cited in this paper were destructive, designed to extract some or all of the gas from the bulk sample, rather than targeting individual inclusions. The extraction of fluid inclusions from rocks is a challenging process as gas concentrations such as CH_4_ can be altered during mechanical crushing via adsorption, contamination or chemical reactions at high temperatures^3^.

Our crush-fast scan method, alongside the cited static crush method^1^, has the advantage of low blank value production, however there is little control over the minimum grain size produced and therefore the gas yield is relatively low. The crushing methods have minimal or no restriction on the size of the inclusion opened or its gas composition. However, during crushing adsorption can be a significant issue, as well as gas production due to the reaction between released gases, silicate surfaces in the crushed material and possible hydrogen and carbon in the walls of the crushing device^3^.

Ball-milling methods are typically associated with significant blank production problems due to the potential adsorption of gases onto freshly ground material, and possible production of gases including hydrogen and methane from the carbon contained in stainless steel that has typically been used for the ball mills^3^. In our study, we used an agate ball mill to try and minimize the known CH_4_ production associated with the use of stainless steel ball mills^3^. We note that quartz and feldspar ‘blanks’ milled in the agate ball mill produced ~100 × less methane than the magnetite and basalt (Supplementary Table 4) – suggesting that the methane produced from the latter were not artefacts. This conclusion was supported by the results of the low energy, low temperature grinding experiments with the magnetite. When normalized to magnetite mass, similar concentrations of methane were produced from mixtures of magnetite and quartz at 0 ⁰C (39.5 ± 17.8 nmol g^-1^) under gentle grinding over 35 days, as produced in the faster, higher temperature agate planetary ball milling (46.3 ± 3.9 nmol g^-1^) (Supplementary Table 6). Note that greater methane was produced from experiments with mixtures with quartz, than with plagioclase, or magnetite alone (Supplementary Table 6). We interpret this as being caused by the different hardness of the minerals. Quartz (7 on Mohr scale) is harder than plagioclase (6 on Mohr scale), hence abrades the magnetite more readily. The low amount of methane production may be because the in starting magnetite grain size was already small (< 63 μm), and may have been close to the terminal grain size of the ball milling process without the addition of larger grains. Overall, we argue that the agate ball mill and low energy grinding experiments together provide realistic upper estimates for the methane contents of the ground minerals/rock. In contrast, the static crush methods, while sensitive when coupled to a mass spectrometer, most likely provide substantial underestimates of total mineral/rock gas content. However, the static method none-the-less provides a sensitive method for comparing the relative gas content of different materials e.g. between terrestrial basalts and SNC meteorites (Supplementary Table 1). Direct comparisons of static and ball-mill crushing techniques using mid-ocean-ridge basalt samples have previously been made^3^. This study also concluded that the ball-mill method provides an efficient way of releasing trapped gases, whereas static crushing in copper tubes is not a very efficient method and produces relatively low gas yields. Supplementary Table 4 demonstrates that the ball-mill method produces at least an order of magnitude more gas compared to static crushing and in some cases there is a three order of magnitude difference in moles of gas released^3^.

To extract methane from our evaporite samples, a dissolution method was used. This method is an efficient way to release gas from samples that should avoid many of the artefacts associated with the use of crushing apparatus. We note that in some cases the salt will not completely dissolve, hence values from this method should in these cases be considered as minimum estimates of gas content.

Finally, the methane in the mudstone/shale samples was extracted via a headspace method^4^ (Supplementary Table 11). The headspace method will most likely underestimate the total amount of methane gas contained within the sediment. A complete method on extraction is given by the original literature^4^ and is outlined below.

Methane was outgassed over 14 days from samples 1–4 cm in diameter. The samples were sealed in gas tight IsoJar containers with septum-fitted lids. The headspace was filled with air and corrected for the 1.8 ppmv methane in the air. Outgassing experiment (not published in Clark et al.) showed shale and limestone materials to have constant methane concentrations after 4 days. The samples were analysed on a continuous flow isotope ratio mass spectrometer. The sample inlet was equipped with both combustion (d13CCH4; ± 0.5‰) and pyrolysis (dDCH4; ± 2‰) gas chromatographs. Calibrated standards, along with peak areas, allowed concentrations to be determination within ± 5%.

**Calculating atmospheric methane fluxes**

Following the methods described, a quantity of methane gas was extracted from the various samples in mols. Most of the samples analysed had a weight of either 0.5 g or 1 g. Where this was not the case the molar value of methane extracted was multiplied accordingly to scale up to mols CH_4_/gram of rock.

Gas data were given in cm^3^ (STP)/gram of rock^3^. The ideal gas law was used to convert these values into mols/gram of rock:

$n=\frac{PV}{RT}$ (1)

Where P is pressure (1 atm), V is the volume of methane, R is the Boltzmann constant and T is the temperature in Kelvin (273.15K). In the dissolution method, methane concentrations in the headspace were corrected for dissolution within water using Bunsen (β) coefficients^5^.

From the moles of gas extracted, the amount of methane in a cubic meter of mineral was calculated using the simple density equation:

$\rho_{mineral}=\frac{m_{mineral}}{V_{mineral}}$ (2)

Solving for V_mineral_ enables the volume of a cubic meter of sample to be obtained. The density of the individual minerals used during the experiment are shown in Supplementary Table 13.

From this we were able to obtain the amount of methane gas in a cubic meter of mineral:

$({\frac{mol}{V})}_{{CH}_{4}}=\frac{{molg}^{-1} {CH}_{4}}{V_{mineral}}$ (3)

Where (mol/V)_CH4_ is in units of mol/g/m^3^.

The methane flux from the mineral samples was then calculated via the following equation:

$$F_{{CH}_{4}}=({\frac{mol}{V})}_{{CH}_{4}}A$$

(4)

Where F_CH4_ is the methane flux (in units of mol/m^2^/Martian year) and A is the abrasion rate (in units of m/Martian year; selection of abrasion rates is discussed below). Therefore from this, the methane flux from the selection of minerals was calculated using any abrasion rate necessary. Methane fluxes were subsequently converted to ppt/m^2^/sol enabling a better comparison to organic breakdown values to be made. The number of molecules in a given column height of Martian atmosphere were first calculated:

$n_{column}=F_{{CH}_{4}}N_{A}$ (5)

Where N_A_ is the Avogadro constant.

Using a column density^6^ of 8.9 × 10^25^ m^2^, which is representative of the amount of gas in a 0.5 km high cross-section of the Martian atmosphere, the methane fluxes in ppt were calculated:

${ppb}_{{CH}_{4}}=\left( \frac{n_{column}}{\rho_{column}} \right)x{10}^{9}$ (6)

Where ρ_column_ is the mean column density^7^. Equation 6 was multiplied by 10^9^ to convert the ratio in a value of ppb.

We calculated gas fluxes from aeolian abrasion for both a) periods of one hour, assuming vertical mixing over a 0.5 km atmospheric height, relevant for short-term (20 min to 1 hour) *in situ* measurements taken by the Curiosity Rover around Gale Crater^8, 9^, and b) 30 sols, assuming vertical mixing over the entire Martian atmospheric column, relevant for the formation of larger scale methane plumes^7^, and matching the duration used for modelling large methane plumes in the latest Global Circulation Models (GCM)^10^. To calculate the time of vertical mixing at 500 m the following equation was used:

$T=\frac{H^{2}}{K}$ (7)

Where H is the height of atmosphere and K is the diffusion rate methane (10^6^ cm^2^/s)^34^. Using 500 m provides a vertical mixing time of 41.67 minutes. Additionally, the Curiosity rover has a filling time for atmospheric gas of 20 minutes (non-enriched) and 2 hours (enriched)^9^, therefore our time of 1 hour for a 500 m atmospheric column seems reasonable.

Eddy diffusion, K, has been estimated to vary between 3 × 10^6^ cm^2^ sec^-1^ to 10^7^ cm^2^ sec^-1^ in the lower 30 km of the Martian troposphere, with lower values likely to be more accurate in the 0 - 20 km range^11^. Additionally, the variation of K will cause a variation in the concentration of methane in the plume. Assuming that the density of the atmosphere is the same for the bottom part of the atmosphere i.e. 0 - 20 km, then a 3 time increase in K will cause the plume to be 3 times more diluted.

**Abrasion rates**

The abrasion rates used for the purpose of Eq. (4) were chosen in accordance with values published in previous literature.

It has been shown that sand dunes in the Nili Patera dune field have surprisingly high sand fluxes, with inferred abrasion rates of approximately 1-10 μm yr^-1^ for flat ground and 10-50 μm yr^-1^ for vertical rock face^12^. Average abrasion rates on the Martian surface would likely be much lower^13^ and could not produce the methane plumes measured by the Curiosity rover and ground-based observations^7^. However, these plumes are formed in local environments where more rapid abrasion rates occur^12^, over shorter time scales. Therefore, the abrasion rate of 50 µm/yr was chosen to represent local high values of erosion in basaltic material. We assumed a tenfold greater rate of abrasion for evaporitic material relative to hard rock^14^. Therefore, an abrasion rate of 500 µm/yr is used when calculating methane fluxes in local environments dominated by softer sedimentary material where sand movement is high.

To obtain a realistic average abrasion rate on the Martian surface over the Amazonian to the present, we use the rate of 0.01 nm/yr^13^ (0.1 nm/yr for evaporites^14^), derived from the Pathfinder site. This value is likely to better represent the daily value of sand movement on the Martian surface as a whole which we can use when investigating the source of the background methane measured by the Curiosity rover.

The abrasion rate of 0.75 µm yr^-1^ was obtained from a scarp retreat rate model^15^. This value represents abrasion rates in the Gale crater region where the land is mostly mudstone, which has similar abrasion rates to evaporites.

**Supporting Tables**

**Supplementary Table 1. Moles of methane present in analogue Martian basalt and Martian meteorite samples. Gas was extracted via a static crushing method^1^ which has an associated typical error of 3.62×10^-11^nmol/g.**

| **Locality** | **Age** | **Amount of CH_4_ released (nmol/g)** |
| --- | --- | --- |
| Kilauea, Hawaii, USA^16^ | Recent | 2.73x10^-3^ |
| Crawton, UK^17^ | Devonian | 1.29x10^-1^ |
| Crawton, UK (weathered)^17^ | Devonian | 1.95x10^-2^ |
| Rejkavik, Iceland basalt sand | Recent | 4.09x10^-3^ |
| Loch Beag, UK basalt sand^18^ | Recent | 5.74x10^-4^ |
| Helen’s Bay, UK (shocked)^19^ | Ordovician | 6.08x10^-3^ |
| Helen’s Bay, UK (shocked)^19^ | Ordovician | 9.39x10^-3^ |
| Lake Myvatn, Iceland^20^ | Recent | 3.47x10^-3^ |
| Lake Myvatn, Iceland (shocked)^20^ | Recent | 9.27x10^-3^ |
| Lake Myvatn, Iceland (unshocked)^20^ | Recent | 1.38x10^-2^ |
| Easdale, UK (shocked)^21^ | Devonian | 3.20x10^-2^ |
| Easdale, UK (unshocked)^21^ | Devonian | 8.59x10^-2^ |
| Glen Drynoch, UK^22^ | Palaeocene | 8.02x10^-3^ |
| Hanrånge, Gavle, Sweden^23^ | Palaeoproterozoic | 6.96x10^-3^ |
| Michigan, USA^24^ | Mesoproterozoic | 3.08x10^-3^ |
| Helen's Bay, UK^19^ | Ordovician | 2.27x10^-1^ |
| Helen's Bay, UK^19^ | Ordovician | 3.90x10^-2^ |
| Kinghorn, UK^25^ | Carboniferous | 1.49x10^-2^ |
| Portree, UK (weathered)^22^ | Palaeocene | 4.61x10^-3^ |
| Newton Crommelin, UK^26^ | Palaeocene | 4.84x10^-3^ |
| Keills Jetty, UK^27^ | Neoproterozoic | 1.30x10^-2^ |
| Keills Jetty, UK^27^ | Neoproterozoic | 3.69x10^-3^ |
| Giant's Causeway, UK^28^ | Palaeocene | 5.13x10^-4^ |
| Stonehaven, UK^17^ | Devonian | 2.30x10^-3^ |
| Nefyn, UK^29^ | Neoproterozoic | 3.12x10^-3^ |
| Schreiber, Ontario, Canada^30^ | Archean | 6.45x10^-3^ |
| Lonar Crater, India^31^ | Recent | 9.36x10^-4^ |
| Nakhlite MIL03346.205* |  | 2.03x10^-2^ |
| Nakhlite NWA5790* |  | 1.04x10^-2^ |
| Zagami (UWO)* |  | 6.66x10^-2^ |
| LA002 (UWO)* |  | 1.31x10^-2^ |
| Nakhla (OWO)* |  | 3.53x10^-2^ |

(*) – Martian meteorite samples^1^

**Supplementary Table 2. Methane release from Martian meteorite samples during crushing experiments during previous experiments^1^ which has an associated typical error of 3.62×10^-11^nmol/g.**

| **Sample** | **Amount of CH_4_ released (nmol/g)** |
| --- | --- |
| LA002 | 6.91x10^-2^ |
| Nakhla | 1.11x10^-1^ |
| NWA5790 | 3.53x10^-2^ |
| Mil 03346 | 6.87x10^-2^ |
| Zagami | 2.15x10^-1^ |
| Y000749 | 1.37x10^-1^ |
| Murchison | 1.58x10^-1^ |
| Tagish Lake | 2.66x10^-2^ |

**Supplementary Table 3. Materials used in agate ball mill method**

| **Sample** | **Source** | **Starting grain size** |
| --- | --- | --- |
| Icelandic basalt | Eyjafjallajökull volcano, sampled in 2014 | 250 μm to 1 mm |
| Magnetite | Bradstäd Mine, Southern Norway (from geologysuperstore.com) | < 63 μm |
| Quartz | Quartz points from Madagascar (from geologysuperstore.com) | 250 μm to 1 mm |
| Plagioclase feldspar | Southern Norway (from geologysuperstore.com) | 250 μm to 1 mm |

**Supplementary Table 4. Total amount of methane released from samples using the gas-tight agate ball mill method.**

| **Sample** | **Amount of CH_4_ released (nmol/g)** |
| --- | --- |
| Magnetite | 46.3 ± 3.9 |
| Icelandic basalt | 31.4 ± 1.4 |
| Quartz | 0.39 ± 0.26 |
| Plagioclase feldspar | 0.50 ± 0.38 |
| Average all quartz and plagioclase (blanks) | 0.5 |
| Magnetite - average blank | 45.8 ± 3.9 |
| Icelandic basalt – average blank | 30.9 ± 1.4 |

**Supplementary Table 5. Experimental set up for low energy grinding experiments at 0°C**

| **Sample** | **Mass g** | **n** |
| --- | --- | --- |
| Magnetite | 10 | 6 |
| Magnetite | 10 | 6 |
| Magnetite | 10 | 6 |
| Plagioclase Feldspar | 10 | 6 |
| Plagioclase Feldspar | 10 | 6 |
| Plagioclase Feldspar | 10 | 6 |
| Quartz | 10 | 6 |
| Quartz | 10 | 6 |
| Quartz | 10 | 6 |
| Magnetite + Plagioclase | 1 + 10 | 6 |
| Magnetite + Plagioclase | 1 + 10 | 6 |
| Magnetite + Plagioclase | 1 + 10 | 6 |
| Magnetite + Quartz | 1 + 10 | 6 |
| Magnetite + Quartz | 1 + 10 | 6 |
| Magnetite + Quartz | 1 +10 | 6 |
| Blank (empty vial) | 0 | 6 |
| Blank (empty vial) | 0 | 6 |
| Blank (empty vial) | 0 | 6 |

**Supplementary Table 6. Results of low energy grinding experiments at 0°C, with results normalized to a) per gram of total material, and b) per gram of magnetite.**

|  | **Mean nmol g^-1^ of CH_4_** | **stdev** | **n** | **Mean nmol g^-1^ magnetite** | **stdev** | **n** |
| --- | --- | --- | --- | --- | --- | --- |
| Quartz | < 0.01 | < 0.01 | 6 |  |  |  |
| Plagioclase | 0.023 | 0.056 | 6 |  |  |  |
| Magnetite | 0.26 | 0.13 | 6 | 0.26 | 0.13 | 6 |
| Quartz+magnetite | 3.60 | 1.62 | 6 | 39.55 | 17.81 | 6 |
| Plagioclase+magnetite | 1.46 | 0.85 | 6 | 16.07 | 9.31 | 6 |
| Blanks | < 0.01 |  |  |  |  |  |

**Supplementary Table 7. Details of materials and experimental methods for the dissolution method**

| **Sample** | **Source** | **Starting grain size** | **Mass (g) of material** | **Volume of water added (ml)** | **Volume of serum vial (ml)** |
| --- | --- | --- | --- | --- | --- |
| Halite^1^ | Boulby Mine, North Yorkshire | 1-2 mm | 10 | 30 | 100 |
| Potash^1^ | Boulby Mine, North Yorkshire | 1-2 mm | 10 | 30 | 100 |
| Potash^2^ (Sample 7^3^) | Boulby Mine, North Yorkshire | > 2mm | 2.5 | 20 | 30 |
| Potash^2^ (Sample 7) | Boulby Mine, North Yorkshire | 1.18 to 2 mm | 2.5 | 20 | 30 |
| Potash^2^  (Sample 7) | Boulby Mine, North Yorkshire | 0.6 to 1.18 mm | 2.5 | 20 | 30 |
| Potash^2^  (Sample 8) | Boulby Mine, North Yorkshire | > 2mm | 2.5 | 20 | 30 |
| Potash^2^  (Sample 8) | Boulby Mine, North Yorkshire | 1.18 to 2 mm | 2.5 | 20 | 30 |
| Potash^2^  (Sample 8) | Boulby Mine, North Yorkshire | 0.6 to 1.18 mm | 2.5 | 20 | 30 |

^1^ run on GC-FID, using 5.0 grade Ar as carrier gas ^2^ run on GC-PDD, using 5.0 grade He as carrier gas

^3^sample numbers refer to subsamples described in Cockell at al (accepted), Int. J Astrobiology.

**Supplementary Table 8. Methane realised from evaporite samples using the dissolution method. St. dev. calculated on n = 3.**

| **Sample** | **Amount of CH_4_ released (nmol/g)** |  |
| --- | --- | --- |
|  | **mean** | **stdev** |
| Potash^1^ | 0.104 | 0.005 |
| Halite^1^ | 0.024 | 0.014 |
| 7 (potash) > 2mm | 189.9 | 18.0 |
| 7 (potash) 1.18 to 2 mm | 209.2 | 11.2 |
| 7 (potash) 0.6 to 1.18 mm | 194.6 | 7.8 |
| 8 (potash) > 2mm | 208.2 | 25.7 |
| 8 (potash) 1.18 to 2 mm | 213.9 | 19.0 |
| 8 (potash) 0.6 to 1.18 mm | 118.5 | 103.2 |

^1^ run on GC-FID, using 5.0 grade Ar as carrier gas ^2^ run on GC-PDD, using 5.0 grade He as carrier gas

**Supplementary Table 9. Moles of methane present in sylvite and halite samples collected from Boulby mine and celestite samples from** **Rossinure, Ireland. Gas was extracted via a static crushing method^1^ which has an associated typical error of 3.62×10^-11^nmol/g.**

| **Sample** | **Amount of CH_4_ released (nmol/g)** |
| --- | --- |
| Sylvite | 1.75x10^-3^ |
| Sylvite | 6.23 x10^-3^ |
| Sylvite | 1.62 x10^-2^ |
| Sylvite | 1.73 x10^-1^ |
| Sylvite | 4.85 x10^-2^ |
| Sylvite | 6.93 x10^-2^ |
| Sylvite | 1.23 x10^-1^ |
| Sylvite | 1.07 x10^-1^ |
| Sylvite | 8.21 x10^-2^ |
| Sylvite | 1.73 x10^-2^ |
| Sylvite | 5.64 x10^-2^ |
| Sylvite | 9.07 x10^-2^ |
| Sylvite | 2.23 x10^-2^ |
| Halite | 6.06 x10^-1^ |
| Halite | 6.46 |
| Halite | 6.29 |
| Halite | 2.18 x10^-2^ |
| Halite | 2.86 x10^-2^ |
| Halite | 7.67 x10^-4^ |
| Halite | 8.0 x10^-4^ |
| Halite | 4.19 x10^-3^ |
| Halite | 3.47 x10^-3^ |
| Halite | 8.70 x10^-1^ |
| Celestite | 6.85 x10^-3^ |
| Celestite | 1.49 x10^-2^ |

**Supplementary Table 10. Moles of methane extracted from mid-ocean-ridge basalt samples via ball-mill and static copper tube methods^3^.**

| **Sample** | **Gas extraction method** | **Amount of CH_4_ released (nmol/g)** |
| --- | --- | --- |
| CY 74 30-32 (uncorrected for CH_4_ adsorption) | Ball-mill | 2.41 ± 0.054 |
| CY 74 30-32 (corrected for CH_4_ adsorption) | Ball mill | 6.69 ± 0.054 |
| DR 11 1003A | Ball-mill | 1.61 ± 0.0028 |
| DR 11 1001A | Ball-mill | 17.8 ± 0.29 |
| ALV 981-R23 | Ball-mill | 0.759 ± 0.015 |
| P6702-44 | Ball-mill | 5.80 ± 0.10 |
| ALV 979-R13 | Static (copper tube) | 0.232 ± <0.0003 |
| ALV 910-4 | Static (copper tube) | 0.268 ± <0.0006 |
| VULCAN 8-3 | Static (copper tube) | 0.535 ± <0.006 |

**Supplementary Table 11. Moles of methane extracted from Michigan Basin bedrock, Bruce site, Ontario via headspace method^4^.**

| **Sample** | **Gas extraction method** | **Amount of CH_4_ released (nmol/g)** |
| --- | --- | --- |
| Ordovician: Queenston to Cobourg | Headspace | 5x10^5^-9x10^6^ |
| Ordovician: Sherman fall to Gull river | Headspace | 5x10^5^-1.5x10^6^ |
| Silurian | Headspace | 5x10^5^-1.75x10^6^ |

**Supplementary Table 12. Moles of methane extracted from United States gas shale systems^32^.**

| **Sample** | **Amount of CH_4_ released (nmol/g)** |
| --- | --- |
| Antrim | 5.05x10^4^-1.26x10^5^ |
| Ohio | 7.58x10^4^-1.26x10^5^ |
| New Albany | 5.05x10^4^-1.01x10^5^ |
| Barnett | 3.79x10^5^-4.42x10^5^ |
| Lewis | 1.90x10^4^-5.69x10^4^ |

**Supplementary Table 13. Density of minerals and rocks used during the experiment.**

| Mineral Type | Density (g/cm^3^) |
| --- | --- |
| Basalt† | 3.00 |
| Nahklites^33^ | 3.15 |
| Shergotlites^33^ | 3.10 |
| Magnetite* | 5.21 |
| Halite* | 2.16 |
| Polyhalite* | 2.76 |
| Sylvinite* | 1.99 |
| Potash† | 1.28 |
| Shale† | 2.68 |

(*)<http://www.webmineral.com/>

(†)<https://www.aqua-calc.com/>

**Supporting references**

1. Blamey, N.J.F., et al. Evidence for methane in Martian meteorites. *Nat. Comms.* **6**, 7399 (2015).

2. Iversen, J.D. and Rasmussen, K. R. The effect of wind speed and bed slope on sand transport. *Sedimentology* **46**, 723-731 (1999).

3. Welhan, J.A. Methane and hydrogen in mid-ocean-ridge basalt glasses: analysis by vacuum crushing *Canadian Journal of Earth Sciences* **25**, 38-48 (1988).

4. Clark, I.D., et al. Paleozoic-aged microbial methane in an Ordovician shale and carbonate aquiclude of the Michigan Basin, southwestern Ontario. *Organic Geochemistry* **83-84** 118-126 (2015).

5. Capasso, G. & Inguaggiato, S. A simple method for the determination of dissolved gases in natural waters. An application to thermal waters from Vulcano Island. *Applied Geochemistry* **13**, 631-642 (1988).

6. Krasnopolsky, V.A. Uniqueness of a solution of a steady state photochemical problem: Applications to Mars. *Journal of Geophysical Research: Planets* **100**, 3263-3276 (1995).

7. Mumma, M.J., et al. Strong Release of Methane on Mars in Northern Summer 2003. *Science* **323**, 1041-1045 (2009).

8. Webster, C.R., et al. Background levels of methane in Mars’ atmosphere show strong seasonal variations. *Science* **360**, 1093-1096 (2018).

9. Webster, C.R., et al. Mars methane detection and variability at Gale crater*.* *Science*  **347**, 415-417 (2015).

10. Holmes, J.A., Lewis, S. R. & Patel, M. R. Analysing the consistency of martian methane observations by investigation of global methane transport. *Icarus* **257**, 23-32 (2015).

11. Krasnopolsky, V.A. Vertical distribution of water vapor and mars model lower and middle atmosphere*. Icarus* **37**, 182-189 (1979).

12. Bridges, N.T., et al. Earth-like sand fluxes on Mars. *Nature* **485**, 339 (2012).

13. Golombek, M.P. & Bridges, N. T. Erosion rates on Mars and implications for climate change: Constraints from the Pathfinder landing site. *Journal of Geophysical Research: Planets* **105**, 1841-1853 (2000).

14. Chojnacki, M., Banks, M., Urso, A. Wind‐Driven Erosion and Exposure Potential at Mars 2020 Rover Candidate‐Landing Sites. *Journal of Geophysical Research: Planets* **123**, 468-488 (2018).

15. Farley, K.A., et al. In Situ Radiometric and Exposure Age Dating of the Martian Surface. *Science* **343**, 6169 (2014).

16. Wright, T.L. Chemistry of Kilauea and Mauna Loa lava in space and time. *Professional Paper* (1971).

17. Stephenson, D. & Thomas, L. P. Caledonian igneous rocks of Great Britain. *Joint Nature Conservation Committee* **17**, (1999).

18. Perillo, G.M.E., et al. Coastal wetlands: An Integrated Ecosystem Approach. *Elsevier, Amsterdam* (2009).

19. Craig, L.E. Stratigraphy in an accretionary prism: the Ordovician rocks in North Down, Ireland*.* *Transactions of the Royal Society of Edinburgh: Earth Sciences* **74**, 183-191 (1984).

20. Hauptfleisch, U. & Einarsson, Á. Age of the Younger Laxá Lava and Lake Mývatn, Northern Iceland, Determined by AMS Radiocarbon Dating. *Radiocarbon* **54**, 155-164 (2012).

21. Browne, M.A., Smith, R.A. Aitken, A.M.A. Stratigraphical framework for the Devonian (Old Red Sandstone) rocks of Scotland south of a line from Fort William to Aberdeen. *British Geological Survey, Research Report,* **RR/01/04,** (2002).

22. Anderson, F.W. & Dunham, K.C. The geology of Northern Skye. *Memoirs of the Geological Survey (Geological Survey of Northern Ireland)* HMSO, Edinburgh, (1966).

23. Ogenhall, E. Plate tectonic settings of the Svecofennian Palaeoproterozoic volcanic rocks at Hamrånge and Loos, south central Sweden, based on geochemical data. *GFF* **129**, 211-226 (2007).

24. Nicholson, S.W. & Shirey, S.B. Midcontinent rift volcanism in the Lake Superior Region: Sr, Nd, and Pb isotopic evidence for a mantle plume origin. *Journal of Geophysical Research: Solid Earth* **95**, 10851-10868 (1990).

25. Browne, M.A.E., et al. Geology of the Kirkcaldy District: A Brief Explanation of the Geological Map Sheet 40E Kirkcaldy. *British Geological Survey* (1999).

26. Eyles, V.I. The composition and origin of the Antrim laterites and bauxites. *Memoirs of the Geological Survey (Geological Survey of Northern Ireland),* HMSO, Belfast, (1952).

27. Gower, P.J. The Dalradian rocks of the west coast of the Tayvallich peninsula. *Scottish Journal of Geology* **13**, 125-133 (1977).

28. LYLE, P. & Preston, J. Geochemistry and volcanology of the Tertiary basalts of the Giant's Causeway area, Northern Ireland. *Journal of the Geological Society* **150**, 109-120 (1993).

29. Maruyama, S., Kawai, T., Windley, B.F. Ocean plate stratigraphy and its imbrication in an accretionary orogen: the Mona Complex, Anglesey–Lleyn, Wales, UK. *Geological Society, London, Special Publications* **338**, 55-75 (2010).

30. Polate, A., Kerrich, R., & Wyman, D.A. The late Archean Screiber-Hemlo and White River-Dayohessarah greenstone belts, Superior Province: collages of oceanic plateaus, oceanic arcs, and subduction-accretion complexes. *Tectonophysics* **289**, 295-326 (1998).

31. Fredriksson, K., et al. Lonar Lake, India: An Impact Crater in Basalt. *Science* **180**, 862-864 (1973).

32. Curtis, J.B. Fractured Shale-Gas Systems. *AAPG Bulletin* **86**, 1921-1938 (2002).

33. Britt, D.T. & Consolmagno, G.J. Meteorite porosities and densities: A review of trends in data. *35th Linar and Planetary Science Conference* **2108,** (2004).

34. Krasnopolsky, V.A., Maillard, J. P., Owen, T.C. Detection of methane in the martian atmosphere:

Evidence for life? *Icarus,* **172**, 537-547 (2004).
